# Satellite tracking reveals a new migration route of black-necked cranes (*Grus nigricollis*) in Qinghai-Tibet Plateau

**DOI:** 10.7717/peerj.9715

**Published:** 2020-08-19

**Authors:** Ye Wang, Chunrong Mi, Yumin Guo

**Affiliations:** 1 Beijing Forestry University, School of Ecology and Nature Conservation, Beijing, China; 2 Chinese Academy of Sciences, Institute of Zoology, Beijing, China

**Keywords:** Black-necked crane, Migration route, Qinghai-Tibet Plateau, Satellite tracking

## Abstract

**Background:**

The black-necked crane (*Grus nigricollis*) is a vulnerable species and the only species that lives in the plateau. Five migration routes of different populations have been identified, but for cranes wintering in Nyingchi Prefecture, Tibet, the migration route and breeding/summering area are still unknown. The aim of this study was to investigate the spatio-temporal migration patterns of black-necked cranes in this area and to identify important areas for conservation.

**Methods:**

In 2016, we fitted seven black-necked cranes in Nyingchi with GPS-GSM satellite transmitters to record their migration routes. We used ArcGIS 10.2 to visualize important stopover sites and the ‘ggplot’ function in R to analyze the migration patterns.

**Results:**

From March 2016 to May 2019, we recorded nine spring migration and four autumn migration tracks from five individuals. Four individuals spent the breeding/summering season in Qinghai Lake, while the other spent the breeding/summering season in the Jinzihai Wetland of Dulan County, Qinghai Province. Detailed spatio-temporal information showed that the spring migration lasted 8.7 ± 4.6 days and covered 1,182.5 ± 90.4 km, while the autumn migration lasted 30 ± 10.6 days and covered 1,455.7 ± 138 km. Basom Lake and the Shazhuyu River were the most important stopover sites during the spring and autumn migrations, respectively. The cranes spent 4.4 ± 3.7 days in Basom Lake and 26.3 ± 10.7 days in the Shazhuyu River. The black-necked cranes mainly migrated during the daytime (>85 % of the fly points), and 81 % (17/21) of all stopover and roosting sites were in the valley or at lakeside swamps. Only 17.7% (516 / 2,914) of the data points for stopover and roosting sites were in protected areas.

**Main conclusions:**

Our study revealed the breeding/summering areas and migration routes of the black-necked cranes wintering in Nyingchi. These results contribute to a better understanding of the annual spatio-temporal migration patterns and the development of conservation plans for this vulnerable species.

## Introduction

Avian migration refers to the regular movement of birds over a certain distance (Newton, 2008). Population management and habitat protection require information on birds’ migration patterns, such as migration routes and stopover sites (Higuchi et al., 2004). Due to the high mobility of birds, there are technical difficulties in bird migration research. With the satellite tracking successfully applied in bird migration studies, it become possible to reveal detailed information on migration patterns (Mi, Møller & Guo, 2018). This technique generates accurate location and time data, enabling ornithologists to infer stopover sites, stopover duration, migration flyway, breeding, and the wintering range (Ma, 2009).

The black-necked crane is the only crane species in the world (among fifteen species) that lives its entire life in the plateau. It is classified as a vulnerable species (VU) by the International Union for Conservation of Nature (IUCN), with a global population of 10,000–10,200 individuals (IUCN, 2019). Black-necked cranes breed in the Qinghai-Tibet Plateau and Ladakh and overwinter in low-altitude areas of the Qinghai-Tibet Plateau, Yunnan-Guizhou Plateau, Bhutan, and southern Tibet in China (Li, 2014). Previous studies used satellite tracking and bird banding to identify five migration routes: (1) the population wintering in the Caohai Nature Reserve, Guizhou Province, and Dashanbao Nature Reserve, Yunnan Province, migrates to the Ruoergai Nature Reserve, Sichuan Province for breeding/summering, with an estimation of 2,600 individuals (Yang et al., 2005; Qian et al., 2009; Liu et al., 2014); (2) the population wintering in the Napahai Nature Reserve, Yunnan Province, migrates to the Longbaotan Nature Reserve, Qinghai Province, and Shaluli Mountain region, Sichuan Province for breeding/summering, with an estimation of 500 individuals (Wu et al., 1993; Liu et al., 2012; Farrington & Zhang, 2013; Luo & He, 2014); (3) the population wintering in eastern Bhutan, migrates to Shenzha County, Tibet for breeding/summering, with an estimation of 200 individuals (Archibald, 2005; Zhang et al., 2015); (4) the population wintering in the Phobjikha Valley, central Bhutan, migrates northwest to Tibet for breeding/summering, with an estimation of 500 individuals (Chamling C., Co D., Yumco Y. and Yumco P., 2020, pers. comm.); and (5) the recently observed population breeding/summering in the Yanchiwan Nature Reserve, Gansu Province, migrates to Linzhou County, Tibet for wintering, with an estimation of 170 individuals (Wang et al., 2020).

Black-necked cranes wintering in Nyingchi Prefecture, Tibet, China, have been reported in previous studies (Tsamchu, Gu & Bishop, 1994; Tsamchu et al., 2008). Researchers also recorded 460 and 527 cranes wintering in this area in 2016 and 2017, respectively (Han & Guo, 2018). However, the breeding/summering area, migration route, and migration patterns of this population are still unknown. The purpose of this study was to: (1) identify the breeding/summering area and migration route of black-necked cranes wintering in Nyingchi, (2) reveal the spatio-temporal migration patterns of this population, and (3) determine the important stopover sites and assess the efficiency of local protected areas.

## Materials & Methods

### Study area

Nyingchi Prefecture (26°52′–30°40″N, 92°09″–98°47″E) is located in southeast Tibet, within the middle and lower reaches of the Yarlung Zangbo River. The average altitude of this area is 3,100 m. Nyingchi Prefecture has a tropical humid and sub-humid climate, high vegetation coverage, rich water resources, and annual precipitation of 650–2,000 mm (Zhang, 2006). A variety of rare wildlife is distributed across this area (e.g., the snow leopard, *Panthera uncia*; black stork, *Ciconia nigra*; and black-necked crane) (Sun, Yang & Zuo, 2003). Previous studies have indicated that Nyingchi is an important wintering area for black-necked cranes (Tsamchu, Gu & Bishop, 1994; Han & Guo, 2018).

### Satellite tracking

In March 2016, seven black-necked cranes were safely captured in Nyingchi Prefecture using a pole trap or a mist net and fitted with solar-powered GPS-GSM satellite transmitters (models HQBP3622 and HQLN0421, Hunan Global Messenger Technology Company) and engraved color rings (Mi, Møller & Guo, 2018). The entire process, from capture to release, was less than 10 min. The HQBP3622 transmitter weighed 22 g and was attached to the back of the birds using a Teflon strip. The HQLN0421 transmitter weighed 40 g and was attached to the left leg, and the inner diameter of the transmitter was 20 mm (Mi, Møller & Guo, 2018; Wang et al., 2020). The data were transmitted through GSM (Global System for Mobile Communication) hourly/every three hours and consisted of longitude, latitude, instantaneous speed, course, altitude, temperature, voltage, and precision. Two individuals failed to send signals due to transmitter failure, but the tracking data of five individuals was successfully received. The weight of the two transmitters was less than 3% of the cranes body weight, and thus is believed to have minimal impact on behavior (Barron, Brawn & Weatherhead, 2010; Bodey et al., 2018).

### Data analysis

The accuracy of the tracking data was divided into five grades: A (within 5 m), B (within 5–10 m), C (within 10–20 m), D (within 20–100 m), and invalid (Wen, Ren & Xing, 2017). In this study, we only used locations categorized as A, B, and C. The migration routes and heat maps were created, and the cumulative migration distance was calculated via the geometry function in ArcGIS 10.2. The start and end of the migration time were defined as the date from which the individuals departed from and arrived at the breeding/summering or wintering sites (Wang et al., 2018). Migration duration was calculated as the time between the individual’s start and end of the migration. Stopover duration was defined the days individual spent at the most important stopover sites (spring: Basom Lake; autumn: Shazhuyu River) for each migration season. Two migration parameters were previously defined by Abrahms et al. (2017) and Buechley et al. (2018): migration straightness (direct distance/cumulative distance) and migration speed (cumulative distance/migration duration). We calculated corresponding migration parameters based on the above definition. Boxplots were used to show the migration patterns (Deng et al., 2019). *P* value was calculated to compare migration parameters between seasons by T test. The sites passed during the migration process involved two time zones, UTC+6 and UTC+7. The local sunrise and sunset times were sourced from an online database (https://richurimo.51240.com/) to determine the daily migration periods. Sunrise and sunset were approximately 07:00 and 20:00 during the spring migration and 08:00 and 19:00 during the autumn migration. Heat maps were used to show the density of points by a density function (Zhao et al., 2014; Perrot et al., 2015). Using the data during migration to draw the heat maps, it can be found that the areas with highly dense of points on the migration route are important stopover sites. Stopover sites were defined as places where a bird stopped for more than two days, while roosting sites were defined as a place where a bird stopped for less than two days (Kölzsch et al., 2015). These points were identified from speeds equal to zero, and fly points were identified by speeds greater than 10 km/h (Mi, Møller & Guo, 2018). The conservation efficiency was determined by the proportion of stopover and roosting site points in protected areas (World Database on Protected Areas: https://www.iucn.org/theme/protected-areas/our-work/world-database-protected-areas). All data are presented as the mean ± SD.

### Ethical note

The study is executed according to Chinese laws on bird capture and handling (Administrative licensing (01401), National Forestry and Grassland Administration, China).

## Results

### Data collation

From 2016 to 2019, 51,280 positions were obtained, of which 49,324 with accuracies A, B, and C were used in this study (details in Table 1). Among the five successfully tracked cranes, three (No. 1, No. 2, and No. 3) were only tracked for one spring migration in 2016. One bird (No. 4) was tracked for three annual cycles from 2016 to 2018, but we only used data before the 2017 spring migration due to missing data. Bird No. 5 was tracked for four spring migrations and three autumn migrations, between 2016 and 2019. In total, nine spring migrations and four autumn migrations were tracked from five individuals.

### Breeding/summering areas and migration patterns

**Figure 1:**
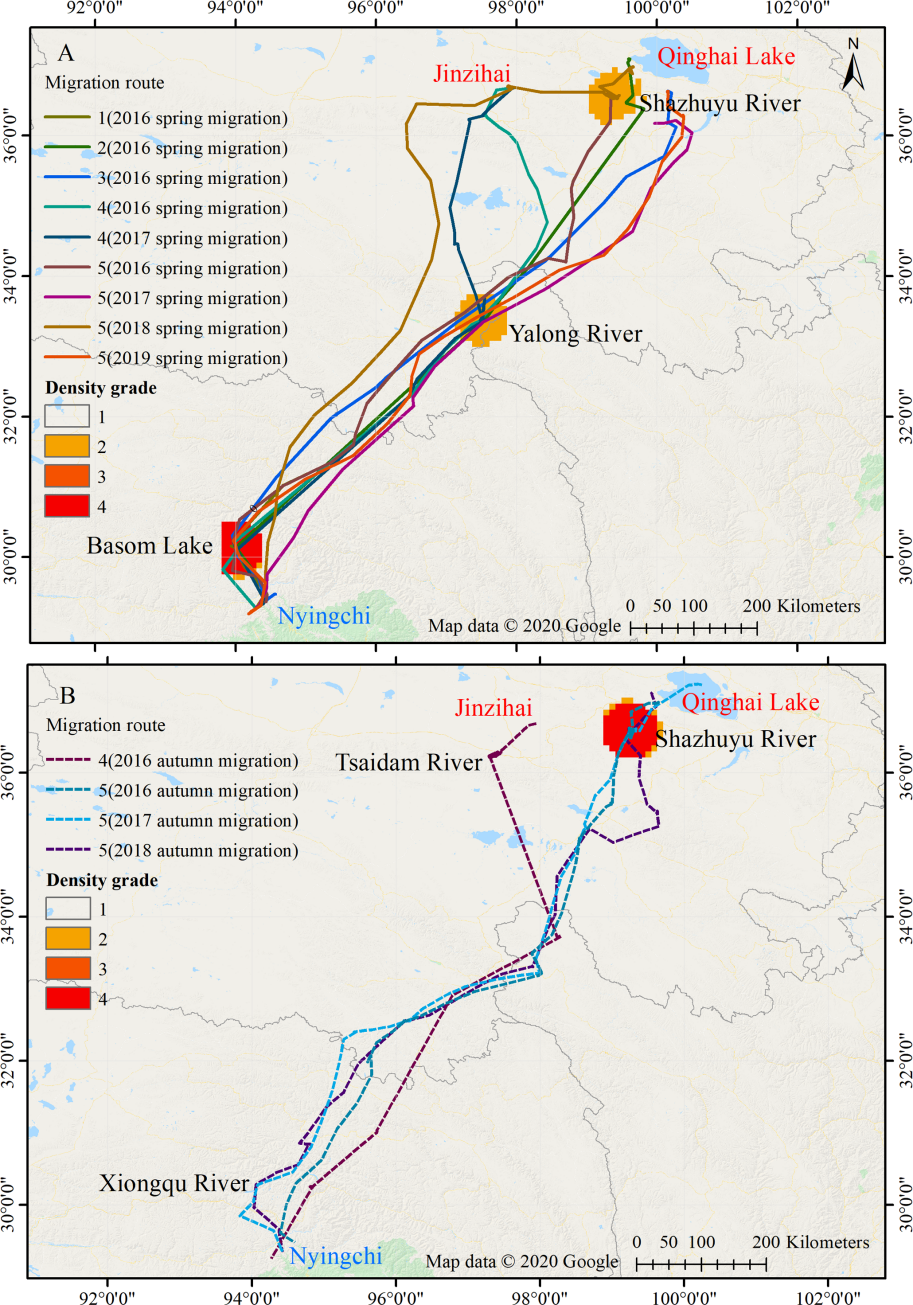
Migration routes of the Nyingchi-Qinghai black-necked crane population. (A) Spring migration route. (B) Autumn migration route. Nyingchi (in Tibet) is the wintering area of the cranes; Basom Lake, Xiongqu River, Yalong River, Tsaidam River, and Shazhuyu River are the stopover sites of the cranes; Qinghai Lake and Jinzihai (in Qinghai province) are the breeding/summering areas of the cranes. The density grade represents the level of point density in the migration routes (Grades 1 to 4, with increasing density). Map data ©2020 Google.

Among the five black-necked cranes wintering in Nyingchi Prefecture, four (No. 1, No. 2, No. 3, and No. 5) spent their breeding/summering season in Qinghai Lake, and the remaining bird (No. 4) spent its breeding/summering season in the Jinzihai Wetland of Dulan County, Qinghai Province (Fig. 1). Their migration routes were highly consistent both intra- and inter-annually in our study (Fig. 1).

**Figure 2:**
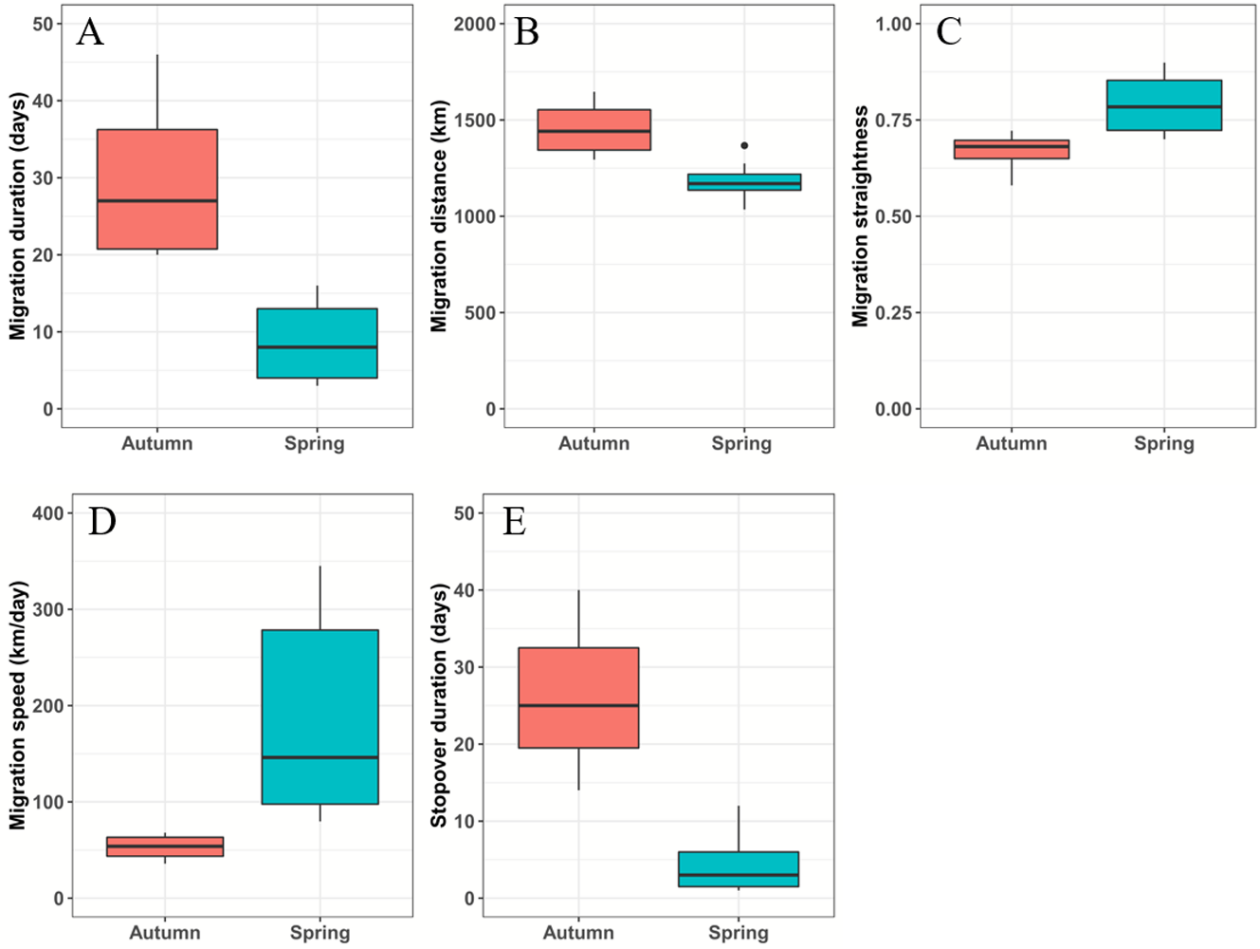
Boxplots of the black-necked crane migration parameters during spring (blue-green) and autumn (red). (A) Migration duration (days). (B) Migration distance (km). (C) Migration straightness. (D) Migration speed (km/day). (E) Stopover duration (days). The spring and autumn stopover durations indicate that cranes stayed at Basom Lake and the Shazhuyu River, respectively.

From March 12 to April 10, the black-necked cranes underwent spring migration, which lasted 8.7 ± 4.6 days and covered a distance of 1,182.5 ± 90.4 km. From September 26 to October 31, the cranes had their autumn migration, which lasted 30 ± 10.6 days and covered 1,455.7 ± 138 km (Table 2; Fig. 2). The straightness metrics differed significantly between the migration seasons (spring: 0.80 ± 0.07, autumn: 0.66 ± 0.05; *p* = 0.009). The migration speed also differed significantly (spring: 181.8 ± 93.2 km/day, autumn: 52.9 ± 12.7 km/day; *p* = 0.027) (Fig. 2).

**Table 1:** Individual information of tracked black-necked cranes wintering in Nyingchi, Tibet.

Crane ID	Status at capture	Tracking period	Number of locations
No. 1	Juvenile	2016.3.7—2016.11.6	5692
No. 2	Adult	2016.3.7—2016.5.26	1806
No. 3	Adult	2016.3.11—2016.10.5	4791
No. 4	Adult	2016.3.10—2018.12.18	9810
No. 5	Juvenile	2016.3.13—2019.5.9	27225

**Figure 3:**
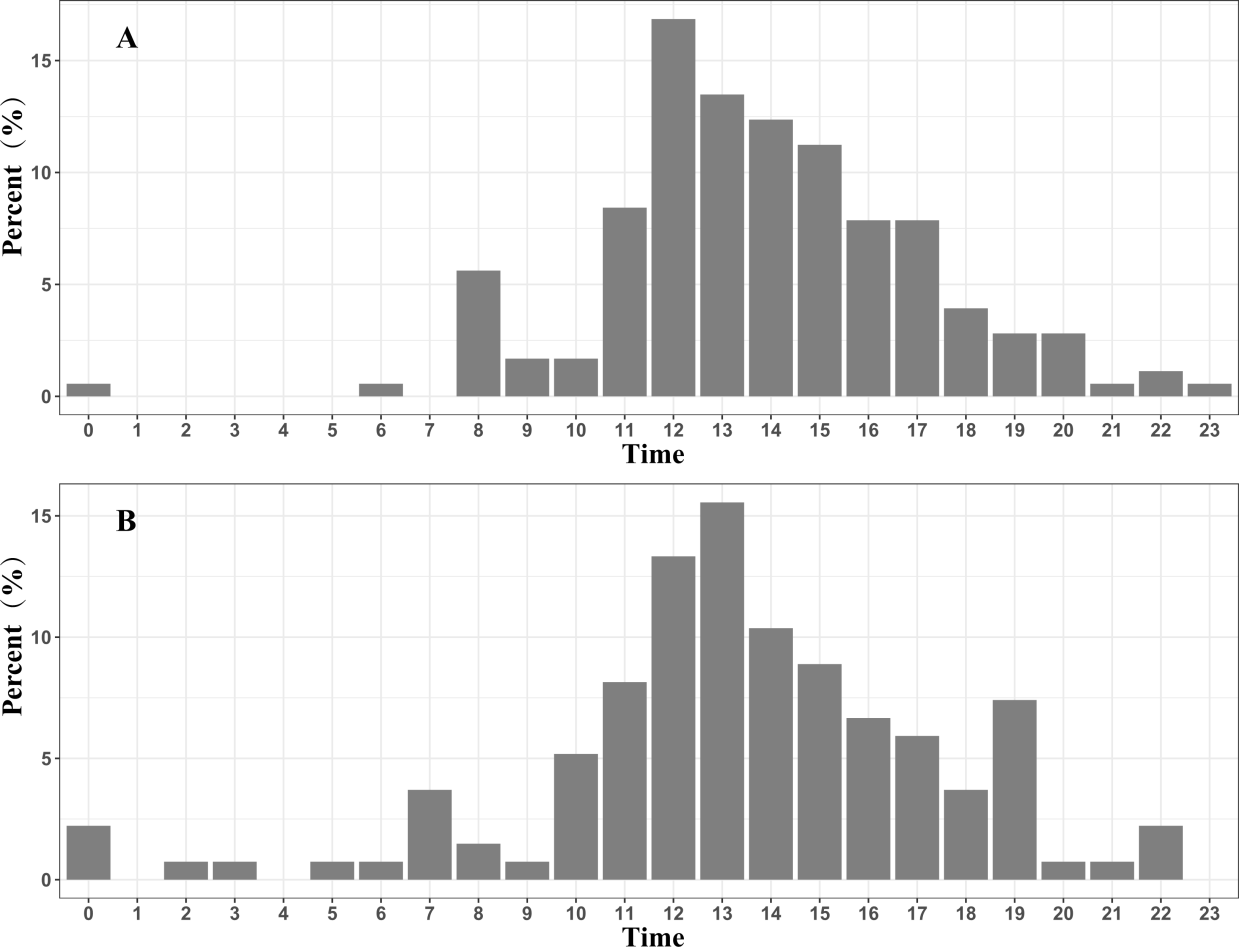
Migration time distribution of the Nyingchi-Qinghai black-necked crane population (shown hourly). (A) Migration time distribution during the spring migration. (B) Migration time distribution during the autumn migration. Data points are the fly points during migration at speeds > 10 km/h.

Approximately 96.6% (172 / 178) of the fly points with a velocity >10 km/h in the spring migration were between 07:00 and 20:00, and 87.4% (118 / 135) of the fly points with a velocity >10 km/h in the autumn migration were between 08:00 and 19:00. These results indicate that the black-necked cranes mainly migrate in the daytime (Fig. 3).

### Stopover sites

Twenty-one stopover and roosting sites were identified for the spring and autumn migrations (Table S1), with three spring stopover sites (Basom, Yalong, and Shazhuyu) and three autumn stopover sites (Shazhuyu, Tsaidam, and Xiongqu Rivers; Fig. 1). The heat maps (Fig. 1) identified Basom Lake as the most important stopover site during the spring migration—there were 686 (38.5%) points located in the lake, and the cranes used it as a stopover site in seven of nine spring migrations (stopover duration = 4.4 ± 3.7 days in March and April; Fig. 2). Basom Lake (29°30′N, 93°36′E) is located in southeast Tibet, within the upper valley of the Ba River. This lake has abundant biological and water resources, with an area of approximately 26.5 km^2^ and an altitude of 3,464 m (Yang, 2019). It is suitable for the subsistence of the black-necked crane. For the autumn migration, the Shazhuyu River was the most important stopover site—there were 1,891 (76.3%) points located in the river, and it was used as stopover site in three of four autumn migrations (stopover duration = 26.3 ± 10.7 days in September and October; Fig. 2). Shazhuyu River (36°33′N, 99°22′E) is located within the Shazhuyu Basin of Gonghe County, Qinghai Province. This river is close to Qinghai Lake and can be used as a stopover site for the black-necked crane during early autumn migration. Seventeen stopover and roosting sites (81%) were located in or near the valley and lakeside swamps, and four were located in meadow swamps (19%; details in Table S1).

**Table 2:** Migration parameters of the Nyingchi-Qinghai black-necked crane population.

Individual	Departure date	Date of arrival and departure at each stopover and roosting site						Arriving date	Migration distance (km)
Spring migration		Basom	Changdu	Yushu	Golog	Haixi	Hainan		
1	2016.4.10	4.10–15	4.15–16	–	4.16–17	–	–	2016.4.17	1169.3
2	2016.4.8	4.8–10	–	4.10–11	–	–	4.11–13	2016.4.13	1135.2
3	2016.4.1	4.1–2	–	4.2–3	–	–	4.3–4	2016.4.4	1114.0
4	2016.3.12	3.12–24	–	3.24–26	–	3.26–27	–	2016.3.27	1274.4
	2017.3.19	3.19–22	–	3.22–30	–	3.30–31	–	2017.3.31	1218.2
5	2016.4.8	4.8–15	4.15–16	–	4.16–17	–	–	2016.4.17	1173.7
	2017.3.22	–	–	3.22–23	–	–	3.23–24	2017.3.24	1035.2
	2018.3.28	–	–	–	3.28–29	3.29–31	3.31–4.10	2018.4.10	1367.3
	2019.4.3	4.3–4	–	4.4–5	–	–	4.5–6	2019.4.6	1155.5

Autumn migration		Tsaidam	Garzê	Yushu	Changdu	Bomê		
4	2016.10.31	10.31–11.9	11.9–10	11.10–11	11.11–12	11.12–20	2016.11.20	1294.0

		Shazhuyu	Hainan	Golog	Garzê	Yushu	Changdu	Basom		
5	2016.9.26	9.26–11.5	–	11.5–6	11.6–7	11.7–9	–	–	2016.11.9	1646.6
	2017.10.12	10.12–11.7	–	11.7–8	11.8–9	11.9–12	–	11.12–13	2017.11.13	1522.1
	2018.10.24	10.24–11.7	11.7–8	11.8–9	11.9–10	11.10–11	11.11–12	–	2018.11.12	1360.1

### Conservation gap

**Figure 4:**
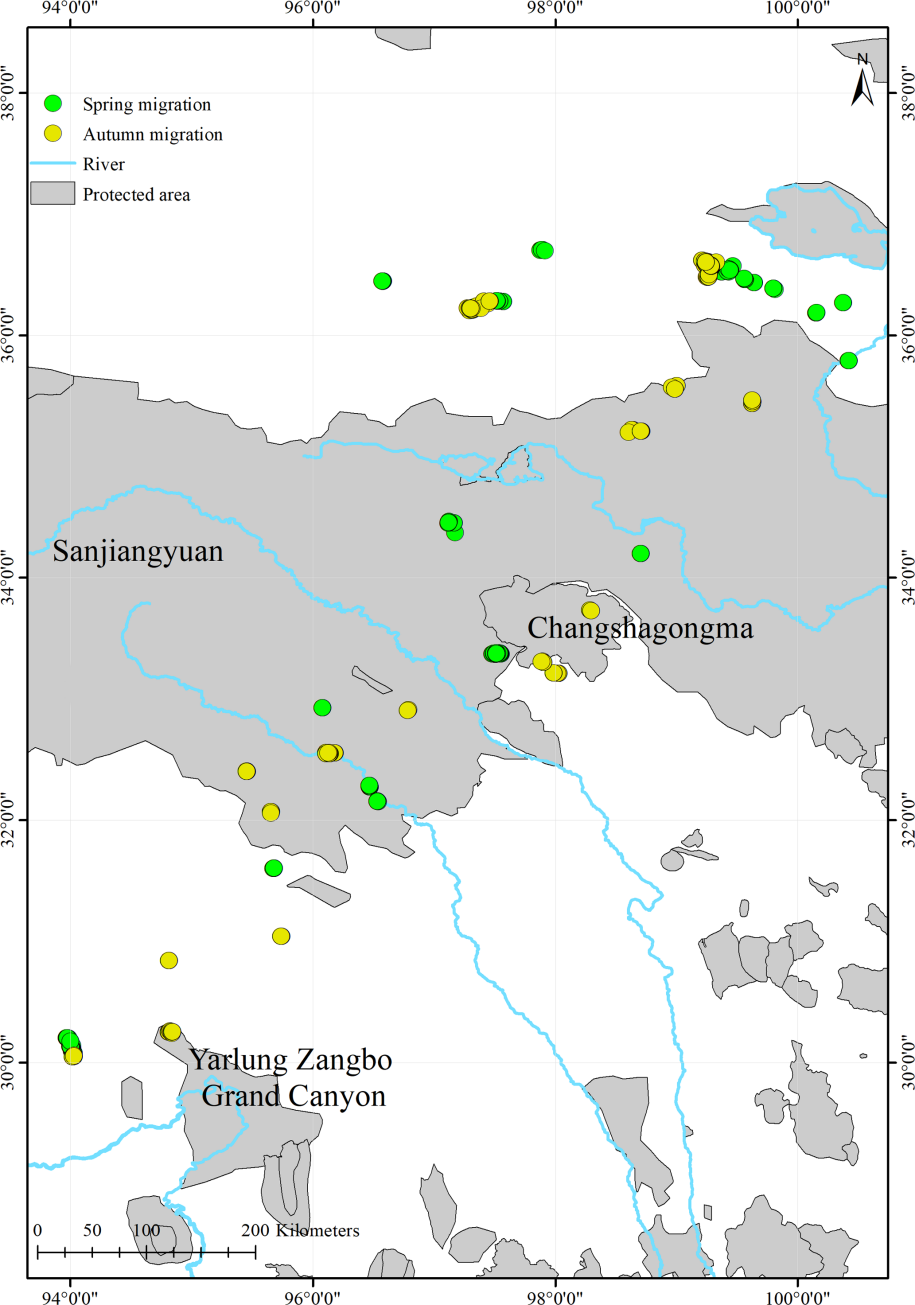
Spatial distribution of the black-necked crane stopover and roosting points during migration and the locations of protected areas within the study areas.

We found that only 17.7% (516/2,914) of the black-necked cranes’ stopover and roosting site points were in protected areas (Fig. 4; Table 3). During migration, the cranes stopped at three nature reserves: the Yarlung Zangbo Grand Canyon Nature Reserve in Tibet (66 points, 12.8%), with a cumulative stopover duration of 9 days; the Sanjiangyuan National Park in Qinghai (432 points, 83.7%), with a cumulative stopover duration of 43 days; and the Changshagongma Nature Reserve in Sichuan (18 points, 3.5%), with cumulative stopover duration of 4 days. The most important stopover sites, Basom Lake and the Shazhuyu River, were not located within nature reserves.

## Discussion

**Figure 5:**
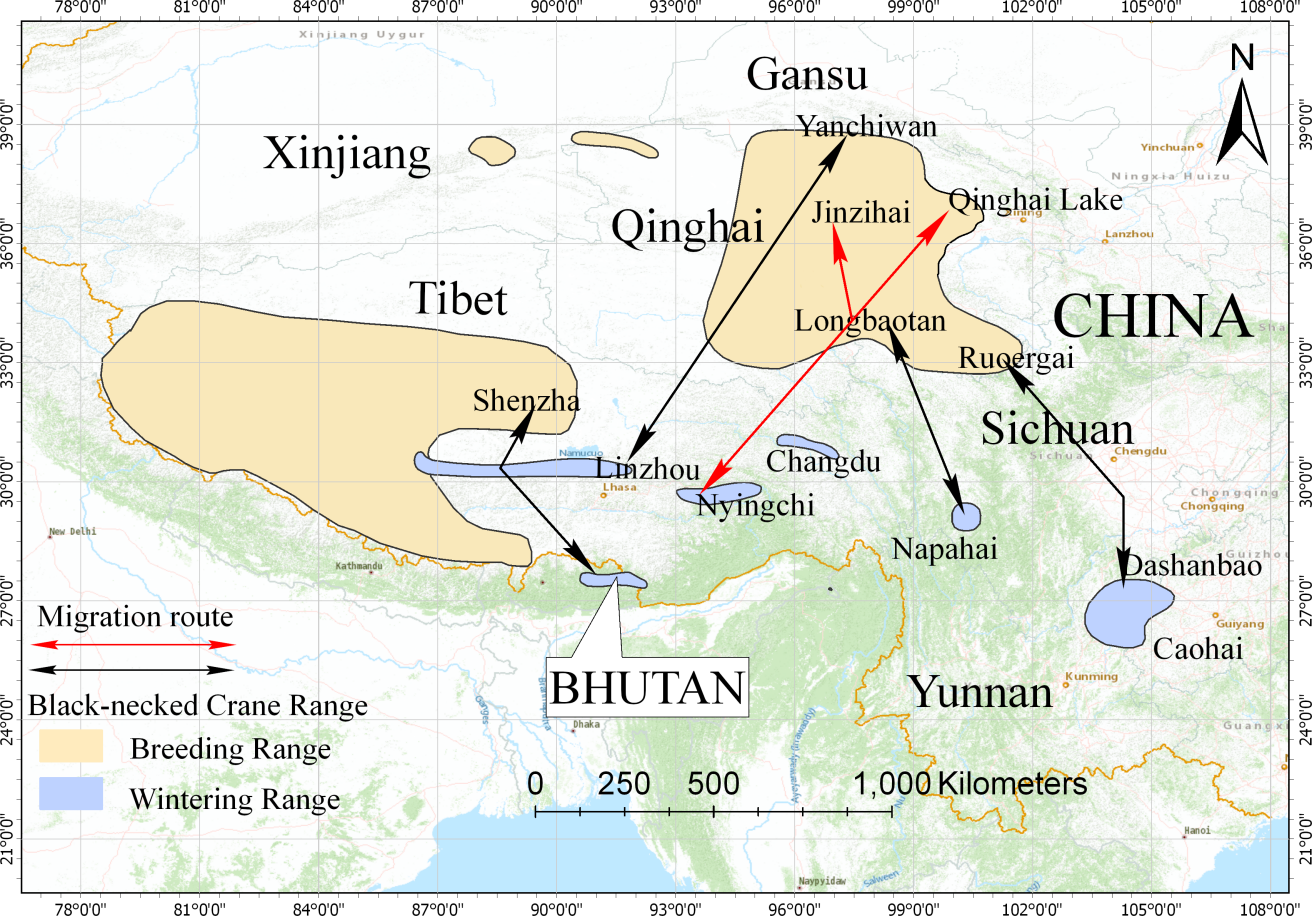
Distribution and migration routes of the black-necked cranes. Areas circled in yellow are breeding areas; areas circled in blue are wintering areas (slightly improved based on the range map from IUCN 2017). Black lines represent the migration routes of the black-necked cranes confirmed by satellite tracking or banding. Red lines represent the migration route of black-necked cranes from this study.

In this study, we used satellite tracking to determine the breeding/summering area and migration route of the black-necked crane population wintering in Nyingchi Prefecture. The tracked individuals had two breeding/summering areas, Qinghai Lake and the Jinzihai Wetland of Dulan County (Fig. 1), the second of which has never been reported. We combined our results with previously published migratory routes to generate the current distribution of black-necked cranes (Fig. 5) (Wu et al., 1993; Archibald, 2005; Yang et al., 2005; Qian et al., 2009; Liu et al., 2012; Wang et al., 2020).

Black-necked crane populations differ in their migration patterns. The average migration duration of the Nyingchi-Qinghai population (spring: 8.6 days, autumn: 30.5 days) is similar to the previously reported values for the Yanchiwan-Linzhou population (autumn: 31.3 days) (Wang et al., 2020), and longer than the Ruoergai-Dashanbao (spring: 5 days, autumn: 5 days) (Qian et al., 2009) and Shaluli-Napahai populations (spring: 1.8 days, autumn: 4.6 d) (Liu et al., 2012). The migration duration of the Bhutan-Shenzha population is unrepresentative, as only one crane was fitted with a satellite transmitter (Archibald, 2005). The average migration distance of the Nyingchi-Qinghai population (spring: 1,183 km, autumn: 1,456 km) was shorter than the Yanchiwan-Linzhou population (autumn: 1,500 km) (Wang et al., 2020), but longer than the Ruoergai-Dachanbao (spring: 651 km, autumn: 694 km) (Qian et al., 2009), Bhutan-Shenzha (spring: 480 km) (Archibald, 2005), and Shaluli-Napahai populations (spring: 256 km, autumn: 219 km) (Liu et al., 2012). Breeding/summering and wintering sites are connected by migration routes (Qin, Chen & Xiang, 2008; Rappole, 2013), and previous studies have shown that altitude is the most important factor affecting crane breeding/summering and wintering habitat selection (Han et al., 2017; Han & Guo, 2018). The topography of the Qinghai-Tibet Plateau is dynamic and can vary greatly within a small range (Liu et al., 2012). These factors may explain why different migration patterns exist among the black-necked crane populations.

The migration distances of several crane species in eastern Asia, including the demoiselle crane (*Anthropoides virgo*; 6,600 km), white-naped crane (*A. vipio*; 2,558 km), and hooded crane (*G. monacha*; 3,000 km), are longer than the black-necked crane (Higuchi et al., 2004; Han & Guo, 2018; Mi, Møller & Guo, 2018). Bird migration in the plateau is challenged by high altitudes and low oxygen (Liu et al., 2018), so soaring birds may choose to shorten the migration distances to reduce mortality rates (Zalakevicius, 2000; Lehikoinen & Sparks, 2010). Therefore, we speculate that it is beneficial for the black-necked cranes to migrate shorter distances, but the underlying advantages require further study.

The stopover and roosting sites of the black-necked cranes were mainly in river valleys (Table S1), and the cranes primarily migrated during the day (Fig. 3). Black-necked cranes are soaring birds, migrating with the assistance of updrafts to save energy (Pirotta et al., 2018). Flying along the river valleys during the daytime could take advantage of air currents, especially in high-altitude regions. Migration stopover sites are connection points between the breeding and non-breeding areas that play an important role in the life cycle of migratory birds (Ma, Li & Chen, 2005). In this study, Basom Lake and the Shazhuyu River were the most important stopover sites during the spring and autumn migrations, respectively (Fig. 1). However, neither of these locations is in protected areas (Fig. 4). Regular patrols of these important sites during the migration season and strengthening animal protection education may be good measures for conservation (Mi, Møller & Guo, 2018).

**Table 3:** Spatial distribution of the black-necked crane stopover and roosting points in protected areas (*n* = 5 cranes).

Individual	Location	Longitude/ latitude (°)	Protected area	Number of points	Percent (%)	Period
1	Golog	98.70/34.20	Sanjiangyuan	15	2.9	Spring
2	Yushu	97.55/33.38	Sanjiangyuan	11	2.1	Spring
3	Yushu	96.14/32.55	Sanjiangyuan	14	2.7	Spring
4	Garzê	98.29/33.73	Changshagongma	4	0.8	Autumn
	Yushu	97.15/33.15	Sanjiangyuan	198	38.4	Spring, Autumn
	Bomê	94.82/30.25	Yarlung Zangbo Grand Canyon	66	12.8	Autumn
5	Hainan	99.63/35.45	Sanjiangyuan	14	2.7	Autumn
	Golog	98.31/34.62	Sanjiangyuan	66	12.8	Spring, Autumn
	Garzê	97.90/33.30	Changshagongma	14	2.7	Autumn
	Yushu	96.10/32.34	Sanjiangyuan	114	22.1	Spring, Autumn
Total				516	100.0	

Between 2017 and 2019, individual No. 5 was a sub-adult and did not breed, so its migration route was less direct (Fig. 1). Zigzag migration routes may increase energy consumption and mortality rates of the black-necked cranes, but sub-adults may also drive population dispersion and find suitable habitats (Zhao et al., 2017; Gao, Mi & Guo, 2019). In 2016 and 2017, researchers recorded 460 and 527 black-necked cranes, respectively, in Nyingchi Prefecture (about 5% of the global population). The proportion of juveniles was 8.64% and 6.64%, respectively (Guo & He, 2017). Combined with our tracking results, we speculate that the population wintering in Nyingchi is stable. The Nyingchi population, together with previously identified crane populations, enriches the population ecology of black-necked cranes (Li, 2014; Wang et al., 2020). These populations appear to be stable, but future declines due to human disturbance or habitat loss may have a huge impact on the survival of the black-necked crane.

## Conclusions

Our study identified the breeding/summering areas and migration route of the black-necked cranes wintering in Nyingchi Prefecture, Tibet, China. These results not only reveal information about this population but also contribute to future conservation plans for this species. However, our study was limited by the fact that only five individuals were tracked, and only three individuals were tracked for one spring migration. More research is needed to validate our results, to explore whether black-necked cranes are loyal to their original migration route, and to investigate potential interactions between different populations.

##  Supplemental Information

10.7717/peerj.9715/supp-1 Supplemental Information 1 Stopover and roosting sites used by the black-necked cranes during migration R. = River, L. = Lake and C. = County.

10.7717/peerj.9715/supp-2 Supplemental Information 2 Migration parameters of each tracking individual The spring and autumn stopover durations indicate that cranes stayed at Basom Lake and the Shazhuyu River, respectively.

10.7717/peerj.9715/supp-3 Supplemental Information 3 Raw data: satellite tracking data during migration of the five black-necked cranes The raw data was used for statistical analysis of the article, and migration period can be found in Table 2.

## Additional Information and Declarations

### Data Availability

The following information was supplied regarding data availability: The raw data are available as Supplemental Files.
