# Supplementary material for: Satellite tracking reveals a new migration route of black-necked cranes (Grus nigricollis) in Qinghai-Tibet Plateau
Source: PeerJ. 2020 Aug 19;8:e9715. doi: 10.7717/peerj.9715 (PMC7443078; doi:10.7717/peerj.9715)
Supplement: Supplemental Information 1 — R. = River, L. = Lake and C. = County. [file peerj-08-9715-s001.docx]

**Table S1: Stopover and roosting sites used by the black-necked cranes during migration.**

R. = River, L. = Lake and C. = County.

| Location | Longitude/latitude (°) | Cumulative stopover (crane/days) | Habitat | Period | Individual  (n) | Location type |
| --- | --- | --- | --- | --- | --- | --- |
| Basom L. | 94.00/30.12 | 32 | Lakeside swamp | Spring, autumn | 5 | Stopover, roosting |
| Xiongqu R. | 94.83/30.25 | 9 | Valley swamp | Autumn | 2 | Stopover, roosting |
| Daqu R. | 95.73/31.03 | 1 | Valley swamp | Autumn | 1 | Roosting |
| Bhutto L. | 95.68/31.60 | 2 | Lakeside swamp | Spring | 2 | Roosting |
| Nangqian C.  Angqu R.  Zhaqu R.  Yushu C.  Ziqu R.  Yalong R.  Mamu R.  Maduo C.  Duoqu R.  Donggeicuona L.  Daheba R.  Chahanwusu R.  Mangla R.  Gengga L.  Tsaidam R.  Shazhuyu R.  Dulan C. | 95.65/32.07  95.45/32.40  96.27/32.43  96.78/32.90  96.08/32.93  97.75/33.30  98.28/33.73  98.70/34.20  97.12/34.45  98.67/35.22  99.63/35.45  98.98/35.57  100.42/35.78  100.15/36.18  97.30/36.32  99.37/36.55  97.95/36.68 | 1  1  8  1  1  11  1  2  1  2  1  1  1  1  12  92  1 | Meadow swamp  Valley swamp  Valley swamp  Meadow swamp  Valley swamp  Valley swamp  Valley swamp  Meadow swamp  Valley swamp  Lakeside swamp  Valley swamp  Valley swamp  Valley swamp  Lakeside swamp  Valley swamp  Valley swamp  Meadow swamp | Autumn  Autumn  Spring, autumn  Autumn  Spring  Spring, autumn  Autumn  Spring  Spring  Autumn  Autumn  Autumn  Spring  Spring  Spring, autumn  Spring, autumn  Spring | 1  1  3  1  1  3  1  2  1  1  1  1  1  1  2  2  1 | Roosting  Roosting  Roosting  Roosting  Roosting  Stopover, roosting  Roosting  Roosting  Roosting  Roosting  Roosting  Roosting  Roosting  Roosting  Stopover, roosting  Stopover, roosting  Roosting |
