# Supplementary material for: Satellite tracking reveals a new migration route of black-necked cranes (Grus nigricollis) in Qinghai-Tibet Plateau
Source: PeerJ. 2020 Aug 19;8:e9715. doi: 10.7717/peerj.9715 (PMC7443078; doi:10.7717/peerj.9715)
Supplement: Supplemental Information 2 — The spring and autumn stopover durations indicate that cranes stayed at Basom Lake and the Shazhuyu River, respectively. [file peerj-08-9715-s002.docx]

**Table S2: Migration parameters of each tracking individual.**

The spring and autumn stopover durations indicate that cranes stayed at Basom Lake and the Shazhuyu River, respectively.

| Crane ID | Year | Migration duration | Migration distance | Migration straightness | Migration speed | Stopover duration |
| --- | --- | --- | --- | --- | --- | --- |
| Spring migration | |  |  |  |  |  |
| No. 1 | 2016 | 8 | 1169.3 | 0.78 | 146.2 | 5 |
| No. 2 | 2016 | 6 | 1135.2 | 0.86 | 189.2 | 2 |
| No. 3 | 2016 | 4 | 1114.0 | 0.85 | 278.5 | 1 |
| No. 4 | 2016 | 16 | 1274.4 | 0.70 | 79.7 | 12 |
|  | 2017 | 13 | 1218.2 | 0.72 | 93.7 | 3 |
| No. 5 | 2016 | 10 | 1173.7 | 0.78 | 117.4 | 7 |
|  | 2017 | 3 | 1035.2 | 0.90 | 345.1 | — |
|  | 2018 | 14 | 1367.3 | 0.72 | 97.7 | — |
|  | 2019 | 4 | 1155.5 | 0.85 | 288.9 | 1 |
| Autumn migration | |  |  |  |  |  |
| No. 4 | 2016 | 21 | 1294.0 | 0.69 | 61.6 | — |
| No. 5 | 2016 | 46 | 1646.6 | 0.58 | 35.8 | 40 |
|  | 2017 | 33 | 1522.1 | 0.67 | 46.12 | 25 |
|  | 2018 | 20 | 1360.1 | 0.72 | 68.0 | 14 |
